# *eNeuro* at Ten: Just Warming Up

**DOI:** 10.1523/ENEURO.0113-24.2024

**Published:** 2024-03-25

**Authors:** Christophe Bernard

**Affiliations:** Editor in Chief, *eNeuro*

Dear friends,

As we approach the tenth anniversary of *eNeuro* at the close of 2024, it strikes me that 10-year-olds are known for their boundless energy and insatiable curiosity. Much like a child at this pivotal age, *eNeuro* is far from settling down. Instead, we are just gearing up for the lively and exciting journey ahead.

Remember when you were ten? Too old to be considered little, but too young to be taken too seriously. Ten-year-olds are notorious for their enthusiasm, their sudden spurts of growth, and their remarkable ability to never sit still. Likewise, *eNeuro* is buzzing with energy and a refusal to remain static.

We have and will continue to plow new ground. After all, *eNeuro* was designed to serve the community of neuroscientists. I believe that science relies on three pillars: the bench work (obviously), the peer review process (we need an external validation of what we are doing), and the publication of our results (which fosters the growth of knowledge). We have made contributions across all three areas.

To help researchers, we have the following:

1. Registered Reports, to evaluate experimental protocols. Acceptance of a protocol will guarantee the publication of the results, even if they are negative. This approach is invaluable, as reviewing the experimental protocol can significantly enhance it, yielding immediate benefits in terms of both resources and time.
2. The possibility to publish negative results, which is crucial for preventing others from needlessly repeating the same experiments.
3. The opportunity to publish (non-)replication studies, which is essential in the current reproducibility crisis.

To assist reviewers and researchers during the review process, we have implemented the following:

1. A double-anonymous review process that reduces biases based on gender, career stage, and country of origin.
2. A consultation process that allows reviewers and the Reviewing Editor to reach a consensus on the manuscript, including any additional experiments that may be required.
3. The review synthesis, which provides authors unified and factual message.
4. Additional statistical review is provided when necessary.

To guarantee the rigor of the published papers:

1. All papers are handled by active neuroscientists, leaders in their field.
2. We offer a broad range of expertise, with Reviewing Editors from all over the world and effective gender balance.
3. Through the SfN Reviewer Mentor Program, we train the next generation of reviewers, thus ensuring the quality of future reviews.

*eNeuro* has undoubtedly influenced the publication landscape in neuroscience, and its leadership in terms of innovation is recognized. I am proud to have been part of its inception and subsequent growth over the past decade. However, there is another major change on the horizon: at the end of 2024, we will be transitioning to a new Editor-in-Chief. As we stand on the threshold of this new era, let us embrace our “inner ten-year-old”—eager, adventurous, and ready to take on the world. At *eNeuro*, turning ten is not about growing up; it is about scaling new heights.

